# ‘*We’re touching the topic, but we’re not opening the book*:’ A grounded theory study of sibling relationships in young people with perinatally acquired HIV

**DOI:** 10.1177/1359105320962271

**Published:** 2020-10-13

**Authors:** Hannah Deakin, Graham Frize, Caroline Foster, Michael Evangeli

**Affiliations:** 1 University of London, UK; 2 St Mary’s Hospital, UK

**Keywords:** communication, coping, family, HIV, Grounded Theory, qualitative methods, siblings, social support, stigma, youth

## Abstract

HIV-related stressors affecting young adults with perinatally acquired HIV (PHIV+) and their siblings include parental and sibling ill-health and death, own ill-health, HIV disclosure, and stigma. Young people with PHIV+ typically share their HIV status with family members. We explored sibling relationships in young people with PHIV+. Ten participants (six females, 17–23 years old) with PHIV+ took part in a semi-structured interview, analysed using Grounded Theory. The data were condensed into three theoretical codes: (1) HIV disclosure in sibling relationship; (2) Patterns of communication about HIV between siblings; and (3) Patterns of coping and support in sibling relationship.

## Introduction

Despite evidence that perinatally acquired Human Immunodeficiency Virus (PHIV+) impacts not only individuals with the virus but the whole family (Ramokolo et al., 2019), there remains a limited understanding of the impact of PHIV+ on sibling relationships. Over the last two decades, medical advances have changed the prognosis of PHIV+ from a terminal diagnosis to a chronic one (Sherman et al., 2000). Young people born with PHIV+ are now living longer, healthier lives and the psychosocial impact of living with PHIV+ on relationships and well-being requires urgent investigation.

Findings in paediatric chronic illness populations (e.g. arthritis and cancer) have shown sibling relationships to be affected by sibling ill-health, illness disclosure and parental differential treatment (Weiss et al., 2001). Unlike many chronic illnesses, PHIV+ also remains a highly stigmatised condition that is transmitted from mother to child. Pressure not to reveal their HIV status outside the family (Michaud et al., 2009) results in young people being more likely to share their status with family members (Lam et al., 2007) and suggests that sibling support could be of particular importance. Although chronic illness literature is helpful in beginning to understand the potential impact of PHIV+ on the sibling relationship (Weiss et al., 2001; Wilkins and Woodgate, 2005), PHIV+ has unique psychosocial components that make it unlike other chronic conditions. Research specific to the sibling relationship in PHIV+ is therefore required to accurately represent and understand the impact of the illness.

Significant HIV-related stressors affecting both PHIV+ and HIV-negative (HIV−) siblings include HIV disclosure, stigma/discrimination, parental ill-health/death and sibling ill-health/death (Malee et al., 2011). PHIV+ young people and their HIV− siblings have been found to have poorer psychological health than their general population peers (Gadow et al., 2010). These additional factors also warrant specific examination of the nature of sibling relationships in PHIV+.

In a review of the literature of mental health and coping in children and families affected by HIV/AIDS, Betancourt et al. (2013) note the benefit of identifying protective processes contributing to resilience in young people with PHIV+, to inform interventions. This may include reinforcing existing familial relationships and strengthening naturally occurring support mechanisms within families (Sharer et al., 2016). Abramowitz et al. (2009) found PHIV+ youth (aged 13–21) received high levels of instrumental support (offering help or assistance in a tangible way) from family members and rated satisfaction with family support higher than that of their friends. The role of siblings in adjustment to living with PHIV+ as a chronic illness has not been a focus of research, despite siblings being found to play an important role in therapy management and care for people with HIV in ethnographic research (Merten, 2016).

This Grounded Theory study aims to explore PHIV+ young people’s experiences of their sibling relationships. The current study is part of a thesis that aimed to develop a model of sibling relationships in young people with PHIV+. This study aimed to develop an understanding of the processes involved in sibling relationships, including how HIV affects the sibling relationship and which aspects of the relationship are perceived as supportive in young adults with PHIV+.

## Method

The study used a qualitative, cross-sectional design. The study was given ethical approval from an NHS Research Ethics Committee, the UK Health Research Authority and Royal Holloway University of London Research Ethics Committee.

### Participants

The sample consisted of 10 participants with PHIV+ with at least one sibling each. Potential participants were required to have knowledge of their PHIV+ status for at least 1 year prior to recruitment. Four male and six female participants aged 17 to 23 years took part in a semi-structured interview. Table 1 outlines participant demographic characteristics.

**Table 1.** Participant characteristics.

Ppt. no.	Sex	Age (years)	Age of paediatric disclosure (years)	Country/region of birth	Ethnicity	Relationship status
1	F	17	12	sub-Saharan Africa	Black African	In a relationship
2	M	19	10	sub-Saharan Africa	Black African	Single
3	F	23	15	UK	Black African	Single
4	F	20	9/10	UK	Mixed (White and Black African)	Single
5	F	21	12	UK	Black African	In a relationship
6	M	23	12	UK	Black African	Single
7	F	23	11/12	sub-Saharan Africa	Black African	In a relationship
8	F	18	12	sub-Saharan Africa	Black African	Single
9	M	21	19/20	sub-Saharan Africa	Black African	Single
10	M	20	8	sub-Saharan Africa	Black African	Single

Table 2 outlines participants’ sibling(s) characteristics, with a particular focus on one ‘identified sibling’.

**Table 2.** Characteristics of participant sibling(s).

Ppt. no.	Identified sibling sex	Identified sibling age (years)	Identified sibling aware of participant’s HIV status (Y/N)	Identified sibling’s own HIV status (Neg/Pos)	Currently living in same household as identified sibling? (Y/N)	Relationship to identified sibling (biological/half)	Total number of siblings
1	F	27	Y	Neg	Y	Biological	2
2	F	34	Y	Pos	N	Biological	3
3	F	16	N	Neg	Y	Biological	2
4	F	24	Y	Neg	Y	Biological	1
5	F	27	Y	Pos	N	Biological	2
6	M	26	Y	Pos	Y	Biological	2
7	F	30	N	Neg	N	Biological	2
8	F	14	Y	Neg	Y	Half	2
9	M	14	Y	Neg	Y	Biological	7
10	F	10	Y	Neg	Y	Biological	1

### Interview guide

An initial version of the interview guide was drafted by the first author in collaboration with the final author, based on existing literature. Participants were first asked to describe their family and were then asked more general questions about their HIV diagnosis (e.g. *How does having HIV affect your life now?*). They were then asked to talk about their sibling relationships and how HIV might impact these (with consideration of factors such as HIV status sharing, levels of support and relationships over time). Participants were initially asked about their relationship with an ‘identified sibling’, and were then asked about other siblings, where relevant. Questions were ordered flexibly, according to participant responses. A draft interview guide was adapted based on feedback from three young people with PHIV+ (all aged 17 years old) from the UK Children’s HIV Association (CHIVA) Youth Committee. Following this consultation, changes were made to the interview guide including simplifying the language and asking about HIV more explicitly.

A second editing process for the interview guide took place after the completion of the first three interviews for this study. In line with Grounded Theory methodology, simultaneous data collection and analysis had revealed gaps in the data, areas of interest and new hypotheses to be explored (Corbin and Strauss, 1990).

### Procedure and analysis

Participants were recruited from two multidisciplinary, inner London NHS HIV clinics with caseloads of between 80 and 120 patients. Participants eligible for the study were screened and approached by their clinician to take part during routine clinic appointments. Written informed consent was then sought by the first author. Ten out of 14 young people approached agreed to take part. Participants were offered a £10 voucher for their time.

Interviews were carried out with each participant individually by the first author in the participant’s HIV clinic, in private consultation rooms. Interviews lasted between 36 and 59 minutes (mean 46 minutes). Interviews were audio recorded using a dictaphone, transcribed verbatim by the researcher and analysed using Grounded Theory (Charmaz, 2014). Grounded Theory was considered the most appropriate qualitative method to suit the research aims, as it lends itself to the study of individual processes and interpersonal relationships, with consideration of the interaction between these and wider social processes (Charmaz, 2014).

Initially, convenience sampling of the target population was employed to advance theory development. After completion of the first six interviews, the researcher hypothesised about the role of gender in patterns of communication in sibling relationships and sought to recruit a final two males through theoretical sampling to explore this further. Theoretical saturation refers to the point at which data collection no longer generates theoretical insights, or reveals new properties of existing theoretical categories (Charmaz, 2014). Dey (1999) argues that emerging categories are only ever suggested by the data, rather than ‘saturated’ by it, and that the term theoretical sufficiency better describes this fit. The specificity of the research aims and sample suggested that theoretical sufficiency would be reached with a relatively small number of participants. The researcher additionally aimed for depth and significance within interviews to provide adequacy of data within a sample size of 10 participants.

Data was coded in three stages: initial coding, focused coding and theoretical coding. The aim of initial coding was to remain open to the possible theoretical directions of the data whilst making sense of the content and staying close to the interview data. The researcher conducted detailed, line-by-line coding of the transcript in this manner, which led to the recognition of emerging focused codes. The researcher identified emerging themes by considering which initial codes held the most ‘analytic power’ (Charmaz, 2014: 140), either due to frequency or significance. These phases of analysis were linked and underpinned by on-going memo-writing. Memos were particularly useful when raising focused codes to conceptual categories and specifying the relationships between them (Charmaz, 2014). A diagrammatic theoretical model was constructed to map out the content and direction of connections between categories.

## Reflexivity

The first author is a female Clinical Psychologist and was a Trainee Clinical Psychologist at the time of the interviews. In 2016 in the UK and Ireland, 50% of young people living with PHIV+ were born abroad and 78% were of Black African ethnicity (CHIPS, 2016). Consideration of the first author’s personal background as an HIV-negative, UK-born, Caucasian woman highlighted differences between this author and participants. Information shared by participants in this study may have been influenced by their assumptions about what the first author may or may not understand about their experiences, because of these differences. Additionally, it was essential to bring awareness to any preconceptions that may originate from the first author’s standpoint as the researcher (Charmaz, 2014), to enhance the credibility of the findings (Mays and Pope, 2000).

With reference to the specific research topic, the first author has a brother and therefore was likely to bring their own assumptions about what a sibling relationship might resemble. To encourage reflection on these issues and the position as the researcher, the first author kept a research diary and regularly discussed relevant thoughts, preconceptions and observations with the final author.

### Quality standards

Quality standards for qualitative research were followed (Elliott et al., 1999; Henwood and Pidgeon, 1992). They included situating the sample (using participant demographic and health information) and providing credibility checks through peer supervision and feedback from the last author on aspects of transcription and coding. Additionally, two coded interview transcripts were analysed by two different researchers experienced in Grounded Theory analysis, allowing for independent verification and validation of emerging themes, categories and models.

## Results

Themes related to the study aims are presented below. The data formed four theoretical codes, however for the purposes of this report, only the three codes relating directly to the sibling relationship are discussed (see Table 3).

**Table 3.** Theoretical codes and focused codes.

PHIV+ disclosure in the sibling relationship	Patterns of communication about PHIV+ between siblings	Patterns of coping and support in the PHIV+ sibling relationship
• Growing up as HIV+ siblings	• Finding ways to talk about HIV	• Feeling normal
• Direct/indirect sibling disclosure	• Times of increased sibling communication	• Valuing the sibling relationship
• Guessed/non-disclosure to sibling	• Keeping the secret	• Sources of support

### PHIV+ disclosure in the sibling relationship

Only one participant had no siblings aware of their HIV status. Four participants’ ‘identified siblings’ became aware of their HIV status from another family member (indirect disclosure) (three from their mother, one from their aunt). Participants were not aware that the sibling was going to be told, and felt a lack of power or control in the decision and process. Participants talked about feeling hurt and annoyed by how their HIV status was shared.

‘*. . .my mum was making us pray so we were praying, and then my mum just randomly came out with it and then my sisters were both, they kind of looked at me and they were both like “what?”. . . it was just really random, like the way my mum just kind of told them. And, I don’t know, like I felt kind of hurt by it, because she didn’t tell me she was gonna tell them. And I would have like wanted to do it properly.*’ (P8)

‘*. . .my sister confronted my mother saying “I know that you and [participant’s name] are keeping something”, so she just like confessed and told her. . . I wasn’t there, which I was annoyed about. Because umm, my doctor kept asking me “does your sister know yet?” and I said “no”, umm, “I’ll find the right time for when I want to tell her”*’. (P4)

There were two experiences of direct disclosure to siblings. Participant one chose to share her status with her half-sibling (sister) and described their relationship as resembling a positive peer relationship.

‘*I told her [. . .] It was kind of scary, but she was very cool about it.*’ (P1)

‘*I told them, yeah, I felt like I needed to tell them because having a doctor tell my sibling something that I’ve got, you’re not really gonna believe it until they actually hear it from the horse’s mouth himself*’ (P9)

Participants who spoke about an ‘identified sibling’ who was also PHIV+, described feeling close to their sibling because they both had HIV, with shared experiences, including medical appointments and medication.

‘*I think we just like started getting closer, I think we realised we had more in common or something, so we just got closer like that.*’ (P5)

‘*. . .we kind of went through the same thing, it was just a case of, when we were kids we had our doctor’s appointments at the same time*’ (P6)

Two participants said that growing up with a PHIV+ sibling allowed them to learn from their older sibling’s experiences of HIV.

‘*I just asked my sister, because obviously she knew more about it, so yeah. . . She was just like, as long as you just take your medicines every day you’ll be fine, like, you’re not gonna die.*’ (P5)

‘*There’s been many occasion when he’s tried to coach me through taking medication*’ (P6)

Two participants had not shared their status with their ‘identified siblings’ and believed that this sibling did not know about their HIV status. A further two participants also wondered if their sibling may have guessed their diagnosis after seeing them take medication daily. None of these participants wanted to share their status with their sibling.

‘*. . .it’s not a conversation I like having. It’s not something I want to bring up.*’ (P6)

‘*I feel like he might have put two and two together, by now. Because he knows that I have to take medication every night, he’s seen me in hospital a few times, so, he definitely knows there’s something different between me and him, but he might not just know what it is*.’ (P6)

### Patterns of communication about PHIV+ between siblings

Communication about PHIV+ was limited between siblings, with eight participants commenting that HIV was not something spoken about in their family.

‘*It’s not something we’ve ever said, “oh, let’s sit down and talk and discuss this”, it’s never happened*.’ (P7)

Being asked limited, direct questions by their sibling about medication and medical appointments allowed their sibling to check in with them without having to engage in deeper discussion, which they appreciated. Participants also commented that their siblings did not refer to HIV specifically, something they favoured.

‘. . .*she really randomly asked how my appointment went. And then I was like “yeah, my viral load is down” and she was like “good”, and then like walked off*” (P4)

“*. . .we don’t talk about it specifically, but they’ll be like “oh, (name), you’re not well, you should take medication” or they’ll like notice something and be like “oh, are you ok?”*’ (P8)

‘. . .*it will probably be “how did the appointment go?” but not be specific and say “how did the HIV appointment go?”. . . so it’s, we’re touching the topic but we’re not opening the book. So, yeah it’s a case of it’s ok, the questions that she’s asking ’cos I’m not thinking about it too much, it’s a quick answer, she knows how I’m feeling, I know how she’s feeling, that’s it, conversation done*.’ (P9)

Four participants discussed never naming HIV with siblings, instead using another word for HIV.

‘. . .*they always referred to it as “the virus,” like from when I was a kid growing up*’ (P4)

‘. . .*we just call it “the club”. So, even when we’re in public we just go “yeah, got to go to the club [. . .] we’ll just be like “oh yeah, I just went like to the club today and you know, same old, same old*’. (P5)

Two participants also referred to making a joke about HIV, which made it easier to talk about together.

‘*Like we just make jokes about it like, we kind of just make fun of ourselves [. . .] If I’m with my sister and it comes up, we just laugh, ’cos it’s just funny. It’s like an inside joke, kind of thing*.’ (P5)

‘. . .*she kinda, I don’t know, takes it, tries to make it seem like light-hearted. . . I don’t know, it’s kinda like, it’s kind of our thing so she makes it seem like, I don’t know, not as bad as it actually is. . . she’s just there, just trying to make jokes out of it. And so, yeah, that kinda helps because, not having to take it seriously all the time*.’ (P8)

Six participants referred to periods of increased HIV communication with their sibling(s). These included the period soon after their sibling had found out about their status and when the participant was unwell. One participant discussed how his brother tries to encourage him to take his medication when unwell because of poor medication adherence.

‘*I think it puts a real burden on the family when I get ill. So, it like, it’s just like, “get on with it”, you know*.’ (P6)

Five participants felt it was best to keep their HIV diagnosis a secret. They were aware that their parent(s) and family did not talk openly about HIV, knowing that it was not something they should tell people about. Participants also spoke about hoping that their sibling would keep their HIV status a secret, with some participants explicitly asking them not to tell anyone.

‘*I guess maybe it’s like having to keep a secret as well. That’s changed my life*’ (P4)

‘. . .*she was like “why didn’t you tell me?” and I said I wasn’t really allowed to tell anyone. . . So, I asked her not to tell people and she said “ah, ok”*’. (P1)

### Patterns of coping and support in the PHIV+ sibling relationship

Nine participants described feeling that HIV had not affected their relationships with family, including their sibling relationships. Alongside the belief that HIV had not changed things, was the notion that participants did not see themselves as being different to others or changed by HIV.

‘. . .*it’s like a normal relationship, we’re there for each other*’. (P3)

‘. . .*we still obviously like get on each other’s nerves and stuff, so that obviously hasn’t changed. So, yeah it’s just kind of normal, the way it always has been*’. (P8)

Reciprocal support between siblings took the form of both emotional support (talking and listening about problems or concerns) and practical support, often unrelated to HIV.

‘. . .*we’re always like a team kind of, in that sense. Whereas, obviously you don’t have that with your friends. We kind of team up when it comes to like, either looking after my [younger] sister or like the house, like maintaining the house or cleaning and stuff*’. (P3)

‘*Whenever I can tell that she’s going through something, I do let her know that I’m here for her. I do little things to help her out when I can tell she’s struggling [. . .] just little things. If she’s been busy the whole day taking care of my nephew I’ll make dinner for us two, so, like that*’. (P1)

Eight participants described reciprocal emotional (non-HIV related) support between siblings. This seemed to mainly be older siblings offering younger siblings emotional support or advice. This emotional support seemed to increase as they got older.

‘*. . .whenever I need to talk to her, she’s always there. She may call an hour later (laughs), but she’s always there if I need to talk to her*’. (P7)

Five participants talked about receiving HIV-related support from their sibling. This included both emotional and practical support.

‘*Yeah, like when I’m sick, she will help look after me. She cooks for me sometimes*’. (P3)

Five participants also referred to relying on themselves to manage difficulties or problems. This seemed to be a default position for many of the participants.

‘*I tend to just keep things in and just go like, ride with it and just go, yeah*’ (P6)

‘*I’ve always been someone who keeps stuff to myself. Works out better somehow*’. (P7)

‘*. . .my problems are for my business*’. (P10)

Emotional HIV-related support from siblings was particularly valued by participants when their sibling was aware of their status, but their friends were not. Five participants had only family members who were aware of their status.

‘. . .*well obviously because she knows about it, so obviously you can like talk to each other about it, but with other people it’s not really, yeah*’. (P5)

All participants described feeling that the sibling relationship was one of the most important relationships in their life.

‘*It’s very important to me because, you know I’ve grown up with her. She used to change my nappies and stuff like that and it’s just something I feel like it should, it’s very important. It should be very important to like a lot of people. And to be close to your siblings, or at least try to be close with them*’. (P1)

‘. . .*she’s my sister, but she’s also kind of like my best friend, in a kind of clichéd way. But, she is because, I don’t know, when I need her she’s there basically. When we need each other we’re there for each other. . . I’m just a lot closer to her than I guess anyone in my life*’. (P8)

‘. . .*with her it’s more important ’cos obviously you have friends, but then like, even if I didn’t have friends I’d still have my sister, kind of thing*’. (P5)

### Model of sibling relationships in young people with perinatally acquired HIV (PHIV+)

**Figure 1.**
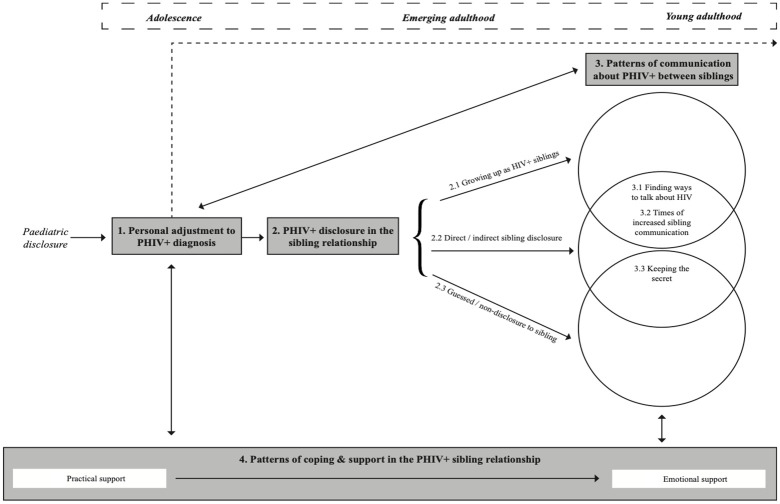
Model of sibling relationships in young people with perinatally acquired HIV (PHIV+).

The aim of the study was to develop a model of sibling relationships in PHIV+. Figure 1 outlines how the main themes are hypothesised to interact in sibling relationships following paediatric disclosure of PHIV+. Focused themes felt to be particularly relevant to the sibling relationship in PHIV+ were included in the model alongside the four theoretical themes.

The model depicts features of the ‘HIV journey’ of a PHIV+ young person post-paediatric disclosure, in relation to aspects relevant to the sibling relationship. The four themes are plotted across a timeline, beginning at paediatric disclosure and running throughout adolescence, emerging adulthood and young adulthood. This timeline provides a context to the experiences of siblings in PHIV+, as illustrated in the main model. The first theme in the centre of the model refers to a period of personal adjustment to PHIV+ diagnosis, directly following paediatric disclosure. This stage is ongoing and includes both an initial response as well as ongoing adjustment into young adulthood (illustrated by the dashed arrow line running across the top of the model). Following this period of personal adjustment, PHIV+ young people experience decisions and events related to disclosure of their HIV status to a sibling. They may have grown up with a PHIV+ sibling and are likely to both be aware of one another’s HIV status. Alternatively, this stage may take the form of either direct/ indirect disclosure or guessed/ non-disclosure. The type and experience of HIV disclosure to sibling(s) affects patterns of communication about PHIV+ between siblings, as does their adjustment to their diagnosis. Certain patterns of communication are more likely to be experienced than others, depending on a sibling’s awareness of their brother/ sister’s HIV status. For instance, siblings who are aware of their brother/sister’s HIV status will find ways to talk to each other about HIV and have times when they talk about it more than others. Siblings who it is believed do not know about their brother/sister’s HIV status or who find out unintentionally are less likely to communicate openly about HIV. The relationships indicated between PHIV+ disclosure in the sibling relationship and communication about PHIV+ between siblings are illustrated in the intersecting circles of the diagram. Levels and types of communication about PHIV+ between siblings also influence patterns of coping and support in the sibling relationship.

Patterns of coping and support with HIV in the sibling relationship are formed primarily post-paediatric disclosure and run alongside the ‘HIV journey’ of young people with PHIV+. Siblings support one another and influence coping strategies at each stage of the model. The evolution of reciprocal support between siblings is observed from practical support when younger, to emotional support when older. The solid arrows in the model show how one theme may feed into and influence another. These relationships between themes may be one-way or bi-directional.

## Discussion

The findings suggest that systemic, relational and individual psychological factors influence young people’s experiences of sibling relationships, when one or both siblings have a diagnosis of PHIV+. Siblings were generally a positive source of support, and there was little evidence of a negative effect of HIV.

Participants described a lack of control around the sharing of their HIV status to siblings. Unplanned disclosure experiences and one’s HIV status being shared by parents are common among families affected by HIV (Kennedy et al., 2010; Proulx-Boucher et al., 2011). The two participants who shared their status with their siblings were in comparatively rare situations, where parents were not in a position of power in relation to onward HIV disclosure (one to a half-sister with a different biological mother, the other after the death of his mother). Power may have been held by parents because disclosing one’s PHIV+ status inevitably leads to disclosure of a parent’s HIV status (Abramowitz et al., 2009). It is also possible that parenting styles with higher levels of control, often found in Black African families (Reitman et al., 2002), may have contributed to a perceived increase in parental authority regarding disclosure.

In general, HIV-related communication between siblings was uncommon. For some, just knowing their sibling was ‘there’ for them was enough and they did not want or feel the need to talk about HIV. At times of ill-health, however, increased support from siblings aware of participants’ PHIV+ status was reported. Such episodes may be considered a ‘centripetal’ force within families, bringing them together between alternative ‘centrifugal’ periods of distance (Combrinck-Graham, 1985).

The type of HIV disclosure (direct vs. indirect) did not seem to affect siblings’ reactions to learning their brother/sister’s HIV status and indirect disclosure did not result in the negative social consequences reported in previous research with an adult (non-perinatal) HIV+ sample (Préau et al., 2015). More generally, participants did not feel they were treated differently by siblings who were aware of their diagnosis. Participants described their sibling relationship as highly valued and close, regardless of whether their sibling knew their status. They tended to seek HIV-related support from siblings or parents and did not feel the need to share their status with individuals outside the family if their sibling was aware and supportive. Five participants had no one in their personal support system outside close family members who knew about their status. This meant that their sibling was potentially the only peer-like figure aware of their HIV status.

### Limitations

Only participants who attended their clinic appointments were approached to take part, which may have led to sampling of a group who were functioning at a higher level or had better levels of sibling support and adjustment in comparison to PHIV+ young people who did not attend. Despite theoretical sampling in the latter stages of recruitment, a small sample size resulted in only two male sibling pairs recruited in this sample (vs six female sibling pairs). Sampling additional male and mixed gender siblings may have provided further insight into levels of communication and support in these relationships, which may differ from female-only siblings (Kim et al., 2006).

It is surprising that cultural factors were not referred to more explicitly by participants. Despite this, it is important to note that all participants were part of an ethnic minority in the UK and that this cultural context may be important with regards to interpreting the findings. A possible explanation for the lack of explicit cultural references in the data is the role of researcher bias. As a white female from Western culture, the first author may have taken an individualistic view and interpretation of participants’ accounts, overlooking any alternative cultural influences in the process. However, participants and their siblings had largely grown up together in the West. It is therefore possible that their/their parent’s culture of origin did not have as much of an influence as it may have done in previous cohorts of PHIV+ young people in the UK who grew up in sub-Saharan Africa. Participants were observed to discuss the impact of Western cultural beliefs (e.g. stigmatising beliefs), which were explored in the analysis.

### Clinical and future research implications

It is hoped that the theoretical model of sibling relationships in PHIV+ produced by this study might be used to inform future therapeutic interventions with this population. The UK CHIVA Standards of Care for Infants, Children and Young People with HIV (Children’s HIV Association, 2017) includes ‘naming of HIV diagnosis to both the infected child and the affected children within the family’ (p. 30) as a key issue. Our study suggests that PHIV+ young people are not often involved in sibling HIV disclosure decisions. Guidance or support for parents about how to involve the PHIV+ young person and facilitate discussion prior to sharing the HIV diagnosis with family members could therefore be beneficial. Supporting PHIV+ young people to share information about HIV with their siblings may also help to encourage communication about HIV in the family home and increase access to supportive sibling relationships. There is currently little guidance about how to facilitate HIV-specific communication within HIV-affected families, although some interventions focus on enhancing familial communication in this population as a way to improve well-being (Bhana et al., 2014). Future research could interview siblings of young people with PHIV+ to gain an alternative viewpoint of the sibling relationship and an insight into coping with having a PHIV+ brother/sister.
